# Supplementary material for: Survival machine learning methods for mortality prediction after heart transplantation in the contemporary era
Source: PLoS One. 2025 Jan 7;20(1):e0313600. doi: 10.1371/journal.pone.0313600 (PMC11706460; doi:10.1371/journal.pone.0313600)
Supplement: S1 File — (DOCX) [file pone.0313600.s001.docx]

**Survival Machine Learning Methods for Mortality Prediction After Heart Transplantation in the Contemporary Era**

| Lathan Liou, MPhil^1,2^ | Lathan.liou@icahn.mssm.edu |
| --- | --- |
| Elizabeth Mostofsky, ScD, MPH^1^ | elm225@mail.harvard.edu |
| Laura Lehman, MD, MPH^1,3,4^ | Laura.Lehman@childrens.harvard.edu |
| Soziema Salia, MD, MPH^1,5^ | soziema@hotmail.com |
| Francisco J. Barrera, MD, SM^1^ | fbarreraflores@hsph.harvard.edu |
| Ying Wei, MD^1^ | yingwei@hsph.harvard.edu |
| Amal Cheema, BA^1,6^ | amalcheema@hsph.harvard.edu |
| Anuradha Lala, MD^7^ | anu.lala@mountsinai.org |
| Andrew Beam, PhD^1*^ | andrew_beam@hms.harvard.edu |
| Murray A. Mittleman, MD, DrPH^1,3,8*^ | mmittlem@hsph.harvard.edu |

^1^Department of Epidemiology, Harvard T.H. Chan School of Public Health, Boston, MA.

^2^Department of Genetics and Genomics Sciences, Icahn School of Medicine at Mount Sinai, New York, NY

^3^Harvard Medical School, Boston, MA

^4^Department of Neurology, Boston Children’s Hospital, Boston, MA

^5^Department of Internal Medicine, Cape Coast Teaching Hospital, Cape Coast, Ghana

^6^Geisel School of Medicine, Dartmouth College, Hanover, NH

^7^Zena and Michael A. Wiener Cardiovascular Institute and Department of Population Health Science and Policy, Mount Sinai, New York, NY, USA.

^8^Division of Cardiovascular Medicine, Department of Medicine, Beth Israel Deaconess Medical Center, Boston, MA

*Authors contributed equally

**Supplementary Appendix**

Below is a short description of the supervised machine learning methods for censored data. The original articles may be consulted for more detailed information about these methods. The Cox Proportional Hazards Model, while not a machine learning algorithm, is included for comparison.

### Cox Proportional Hazards Model

The standard statistical tool for analyzing censored survival data is the Cox proportional hazards model, which evaluates the effect of several variables simultaneously on the time to an event of interest, such as death or the diagnosis of a disease. While it is a robust model, the Cox model does not generalize well to high dimensions, applies only to linear combinations of predictors, and is limited by strict assumptions, most importantly the proportional hazards assumption, that may not hold in the real world. It is included here as a baseline against which to compare the other models.

The Cox model is expressed by the hazard function, which is the instantaneous risk of an event occurring at time t as follows:

*h*(*t*) = *h*_0_(*t*) × *exp*(*β*_1_*x*_1_+*β*_2_*x*_2_+...+*β_p_x_p_*)

where *t* represents the survival time, *h*(*t*) is the hazard function, {*x*_1_,*x*_2_,...,*x_p_*} are the values of the p covariates, {*β*_1_,*β*_2_,...,*β_p_*} are the coefficients that measure the effect of the covariates on the survival time and h_0_(t) is the baseline hazard function, which is unspecified.

The regression coefficients are estimated by maximizing the partial likelihood.

### Penalized Cox Regression

Standard linear regression performs poorly when applied to high-dimensional data. A penalized model that adds a constraint to the equation is often used to overcome this. This constraint reduces or shrinks, the coefficient values towards zero, reducing their variance and ensuring that the less important features have less impact in the model.

The two most common forms of regularization use an L_1_ or an L_2_ penalty constraint. An L_1_ penalty (also known as Lasso regression) is equal to the absolute value of the magnitude of coefficients. It can produce models with fewer coefficients (sparse or parsimonious models) because some coefficients are reduced to zero and therefore eliminated. In this way, the Lasso performs feature selection in addition to fitting the model. An L_2_ penalty (also known as ridge regression) is equal to the square of the magnitude of the coefficients. Ridge regression shrinks all coefficients by the same factor, and none are eliminated, so it does not produce sparse models.

The Lasso suffers from several limitations in that it cannot select more features than the number of samples and where there are correlated features, it tends to select only one from a group indiscriminately. Elastic net regression, a linear combination of the L_1_ and L_2_ penalties of the Lasso and ridge methods, was developed to overcome these limitations and is known to be particularly useful when the number of features is larger than the number of samples.

Lasso, ridge, and elastic net regression have all been extended to the Cox model and are evaluated here.

### Boosted Cox Regression

Boosting is an iterative technique developed in the machine learning community and later adapted to statistical modeling, including survival analysis. It is an ensemble technique that trains weak learners sequentially, where each new model that is added to the ensemble learns from the mistakes of the previous models. Boosting is resistant to overfitting, provided the number of boosting steps is carefully chosen, and it can cope with high-dimensional data.

There are two main approaches to boosting for statistical modeling, including survival analysis – likelihood-based boosting and gradient boosting. Likelihood-based boosting uses base learners that maximize an overall likelihood in each boosting step, selecting only the base-learner which leads to the largest increase in the likelihood. Gradient boosting is equivalent to iteratively re-fitting the residuals of the ensemble model at each step.

In this work, three different boosting algorithms are evaluated – Cox Boost, a likelihood-based boosting algorithm that uses a survival decision tree with a Cox distribution as its base learner, XGBoost Linear, a faster gradient boosting algorithm using Cox models with an applied regularization, and XGBoost Survival Tree, a boosting algorithm using accelerated failure time models to build decision trees.

### Random Survival Forests

Random survival forests are an extension of Breiman's random forest to censored survival data. Random survival forests aggregate the results from many decision trees, each generated from a bootstrap sample of the data. At each node in the random forest, one feature is selected to split on, from a random subset of all features. In a random survival forest, the feature and split point chosen is the one that maximizes the survival difference between daughter nodes i.e., that maximizes the log-rank statistic over all available split points and features.

Random survival forests are becoming more widely accepted as an alternative to the Cox proportional hazards model due to their ability to model complex, non-linear data, handle high-dimensional data, identify interactions and impute missing data naturally. As such, they reduce the tendency for the model to overfit the data and can capture complex interactions between predictors.

**Supplementary Table 1.** List of input predictor variables and their data preparation

| **SRTR Encoding** | **Type*** | **Notes** |
| --- | --- | --- |
| bmi_calculated | cont | Derived from CAN_WGT_KG/ CAN_HGT_CM /CAN_HGT_CM*10000 |
| CARDIAC_ETIOLOGY | cat | Derived from REC_DGN. Refactored to Valvular Heart Disease, Dilated Myopathy, Ischemic Dilated Myopathy, Restrictive Myopathy, Coronary Artery Disease, Other, Idiopathic |
| Dialysis_Baseline | bin | Derived from CAN_DIAL |
| DM_Baseline | bin | Derived from CAN_DIAB_TY and DON_HIST_DIAB |
| WAIT_TIME | cont | Derived from REC_TXT_DT – XCAN_LISTING_DT |
| XCAN_ABO | cat |  |
| XCAN_ACPT_DCD | bin |  |
| XCAN_ACPT_HBC_POS | bin |  |
| XCAN_ACPT_HCV_POS | bin |  |
| XCAN_ACPT_HIST_CAD | bin |  |
| XCAN_CEREB_VASC | bin |  |
| XCAN_EDUCATION | cat |  |
| XCAN_FUNCTIONAL_STATUS | cat |  |
| XCAN_GENDER | bin |  |
| XCAN_PRELIM_XMATCH_REQUEST | bin |  |
| MDRDeGFR_WL | cont |  |
| PREV_MALIG | bin |  |
| status_change | bin | Derived from CAN_INIT_STAT %in% c(1030, 2010, 2020, 2030, 2090) &  CAN_LAST_STAT %in% c(2110, 2120, 2130, 2140, 2150, 2160, 2999) |
| status_upgrade | bin | Derived from CAN_INIT_STAT %in% c(2110, 2120, 2130, 2140, 2150, 2160, 2999) &  CAN_LAST_STAT %in% c(2110, 2120, 2130, 2140, 2150, 2160, 2999) &  CAN_INIT_STAT > CAN_LAST_STAT |
| DON_ABO | cat |  |
| DON_AGE | cont |  |
| DON_ANTI_CMV | bin |  |
| DON_ANTI_HCV | bin |  |
| DON_CAD_DON_COD | bin |  |
| DON_cardiac_arrest | bin | Derived from DON_CARDIAC_ARREST_AFTER_DEATH and DON_NON_HR_BEAT |
| DON_CREAT | cont |  |
| DON_DDAVP | bin |  |
| DON_GENDER | bin |  |
| DON_HGT_CM | cont |  |
| DON_HIGH_CREAT | bin |  |
| DON_HIST_CANCER | bin |  |
| DON_HIST_CIGARETTE_GT20_PKYR | bin |  |
| DON_HIST_COCAINE | bin |  |
| DON_HIST_DIAB | bin |  |
| DON_HIST_HYPERTEN | bin |  |
| DON_HIST_OTHER_DRUG | bin |  |
| DON_INOTROP_SUPPORT | bin |  |
| DON_WGT_KG | cont |  |
| INSURANCE | cat |  |
| MDRDeGFR_B | cont |  |
| REC_A_MM_EQUIV_CUR | cont |  |
| REC_AGE_AT_TX | cont |  |
| REC_B_MM_EQUIV_CUR | cont |  |
| REC_CARDIAC_OUTPUT | cont |  |
| REC_CARDIAC_SURG | bin |  |
| REC_CHRONIC_STEROIDS | bin |  |
| REC_CMV_STAT | bin |  |
| REC_CREAT | cont |  |
| REC_DR_MM_EQUIV_CUR | cont |  |
| REC_EBV_STAT | bin |  |
| REC_ECMO | bin |  |
| REC_FUNCTIONAL_STATUS | cat | Refactored to poor, moderate, normal, and other |
| REC_HBV_ANTIBODY | bin |  |
| REC_HBV_SURF_ANTIGEN | bin |  |
| REC_HCV_STAT | bin |  |
| REC_HIV_STAT | bin |  |
| REC_HLA_ANTIBODY | bin |  |
| REC_HLA_TYP_DONE | bin |  |
| REC_HR_ISCH | cont | amount of time between donor heart procurement and the actual transplant |
| REC_IABP | bin |  |
| REC_INOTROP | bin |  |
| REC_LIFE_SUPPORT | bin |  |
| REC_LIFE_SUPPORT_OTHER | bin |  |
| REC_MED_COND | cat |  |
| REC_MM_EQUIV_CUR | cont |  |
| REC_PCW_MEAN | cont |  |
| REC_PROCEDURE_TY_HR | cat |  |
| REC_PULM_ART_DIAST | cont |  |
| REC_PULM_ART_MEAN | cont |  |
| REC_PULM_ART_SYST | cont |  |
| REC_TOT_BILI | cont |  |
| REC_TXFUS | bin |  |
| REC_VENTILATOR_SUPPORT | bin |  |
| VAD | bin | Derived from REC_VAD_TY |

*cat = categorical, bin = binary, cont = continuous

**Supplementary Table S2.** Training specifications of models

| **Algorithm** | **R package** | **Function** | **Hyperparameters** |
| --- | --- | --- | --- |
| Cox PH model | *survival* | surv.coxph |  |
| Ridge | *glmnet* | surv.cv.glmnet | alpha = 0, nfolds=5, lambda is unsupported in *mlr* package |
| Elastic Net | *glmnet* | surv.cv.glmnet | alpha = 0.5, nfolds=5, lambda is unsupported in *mlr* package |
| Lasso | *glmnet* | surv.cv.glmnet | alpha = 1, nfolds=5, lambda is unsupported in *mlr* package |
| Cox boosting with survival trees | *gbm* | surv.gbm | n.trees = 100  Shrinkage = seq(0.01, 0.1, 10),  Interaction.depth = seq(1, 6, 1) (1 = additive model)  n.minobsinnode = 10  bag.fraction = 0.5 |
| Extreme Gradient Boosting – linear model based | *XGBoost* | Surv.xgboost | booster = "gblinear" |
| Extreme Gradient Boosting – tree model based | *XGBoost* | Surv.xgboost | booster = "gbtree"  gamma: 0  nrounds: 100  max_depth: seq(2, 10, 2)  min_child_weight: seq(0.5, 4.5, 1)  subsample: 0.8  colsample_bytree: 0.8  eta: 0.1 |
| Random Survival Forests | *randomForestSRC* | Surv.randomForestSRC | mtry: sqrt(#features) -> 100  nodesize: 3 -> 25, ntree=1000 |

**Supplementary Table S3.** Cross-validated C-indices

| **Model** | **Post-policy Mean C-index (SD)** | **Pre-policy Mean C-index (SD)** |
| --- | --- | --- |
| XGBoost Linear | 0.615 (0.025) | 0.628 (0.023) |
| Random Survival Forests | 0.612 (0.027) | 0.625 (0.022) |
| Cox Boost | 0.610 (0.028) | 0.627 (0.030) |
| XGBoost Tree | 0.608 (0.024) | 0.611 (0.024) |
| Ridge | 0.608 (0.031) | 0.624 (0.024) |
| Cox | 0.603 (0.031) | 0.606 (0.022) |
| Lasso | 0.516 (0.033) | 0.532 (0.044) |
| Elastic Net | 0.508 (0.025) | 0.514 (0.027) |

**Supplementary Table S4.** Correlated resample paired t-test results

| **Model 1** | **Model 2** | **Post-policy cohort Adjusted *P-value*** | **Pre-policy cohort Adjusted *P-value*** |
| --- | --- | --- | --- |
| Cox | Elastic Net | **0.003** | 0.107 |
| Cox | Cox Boost | 1 | 1 |
| Cox | Lasso | **0.021** | **0.002** |
| Cox | Random Forest | 1 | 1 |
| Cox | Ridge | 1 | 1 |
| Cox | XGBoost Linear | 1 | 1 |
| Cox | XGBoost Tree | 1 | 1 |
| Elastic Net | Cox Boost | **0.001** | 0.055 |
| Elastic Net | Lasso | 1 | 1 |
| Elastic Net | Random Forest | **<0.001** | **0.033** |
| Elastic Net | Ridge | **<0.001** | **0.010** |
| Elastic Net | XGBoost Linear | **<0.001** | **0.023** |
| Elastic Net | XGBoost Tree | **<0.001** | 0.107 |
| Cox Boost | Lasso | **0.016** | **0.001** |
| Cox Boost | Random Forest | 1 | 1 |
| Cox Boost | Ridge | 1 | 1 |
| Cox Boost | XGBoost Linear | 1 | 1 |
| Cox Boost | XGBoost Tree | 1 | 1 |
| Lasso | Random Forest | **0.012** | **<0.001** |
| Lasso | Ridge | **0.001** | **<0.001** |
| Lasso | XGBoost Linear | **0.017** | **<0.001** |
| Lasso | XGBoost Tree | **0.002** | **0.002** |
| Random Forest | Ridge | 1 | 1 |
| Random Forest | XGBoost Linear | 1 | 1 |
| Random Forest | XGBoost Tree | 1 | 1 |
| Ridge | XGBoost Linear | 1 | 1 |
| Ridge | XGBoost Tree | 1 | 1 |
| XGBoost Linear | XGBoost Tree | 1 | 1 |

A Bonferroni adjustment was applied to control the False Discovery Rate at 0.05. Bolded values are statistically significant.

**Supplementary Figure 1.** Heatmaps of the total number of times a) demographic and clinical b) recipient c) candidate d) donor and e) procedural variables were selected by each model in the post-policy cohort

| **A**  **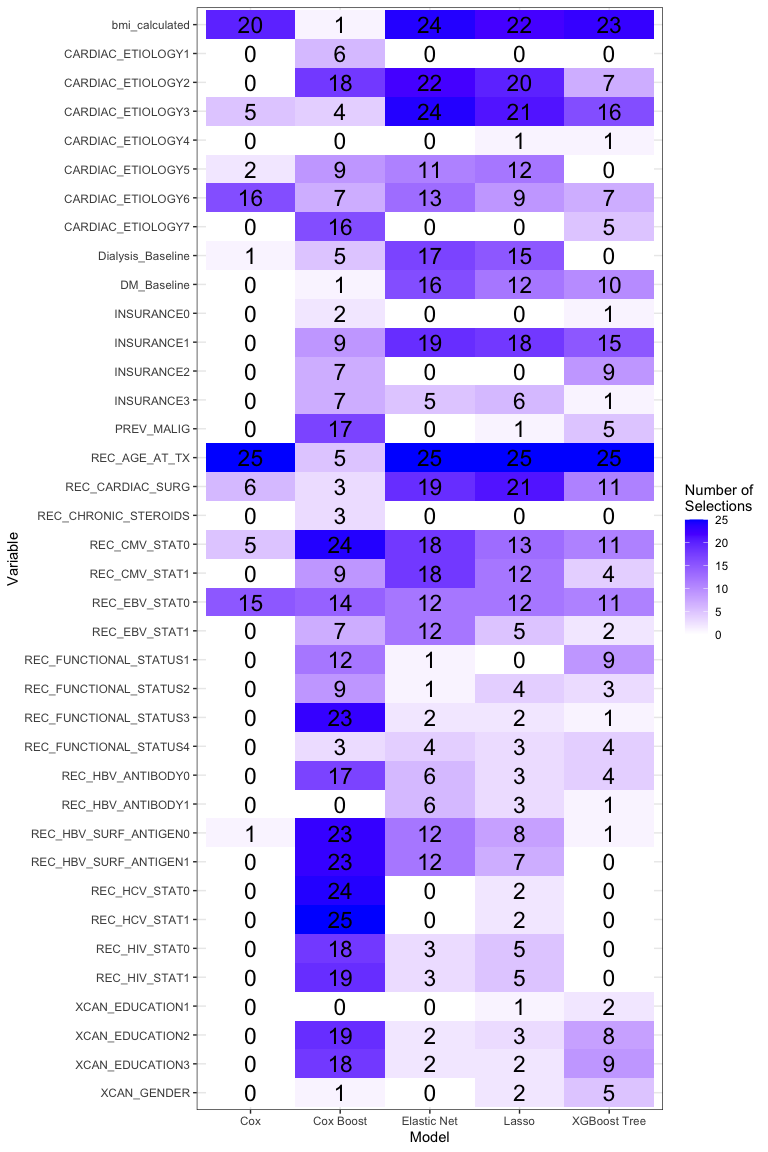** |
| --- |
| **B**  **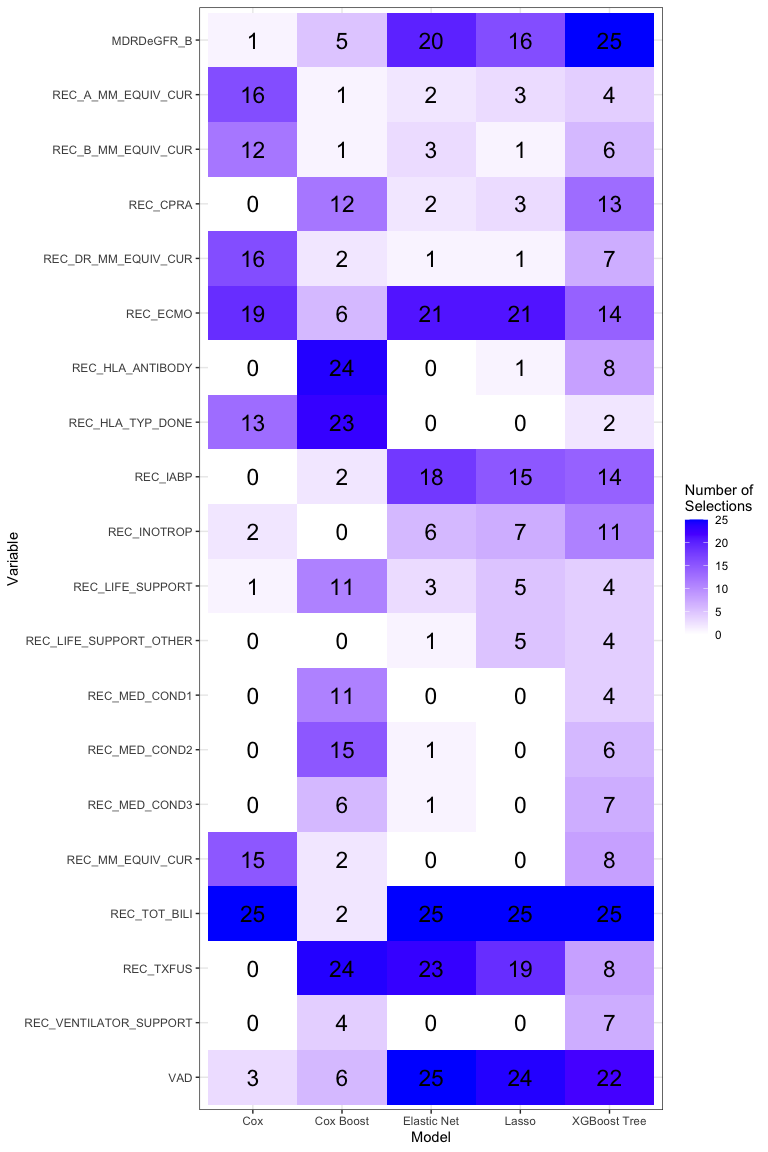** |
| **C**  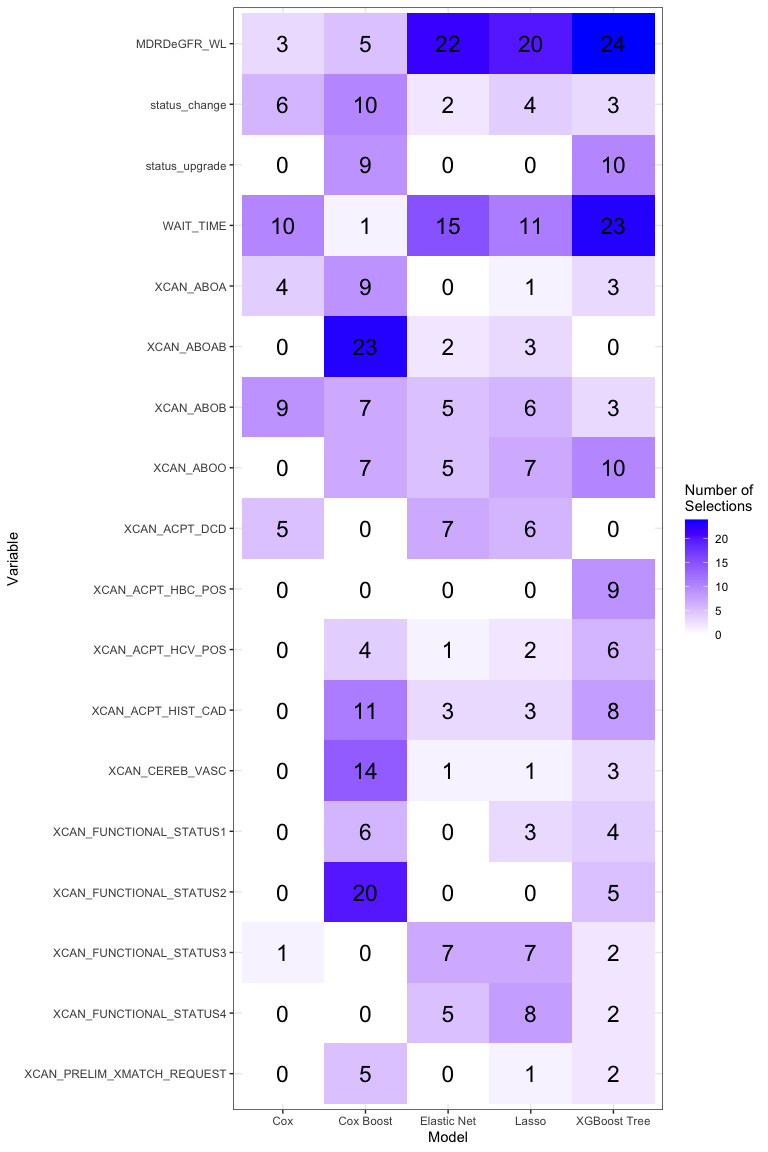 |
| **D**  **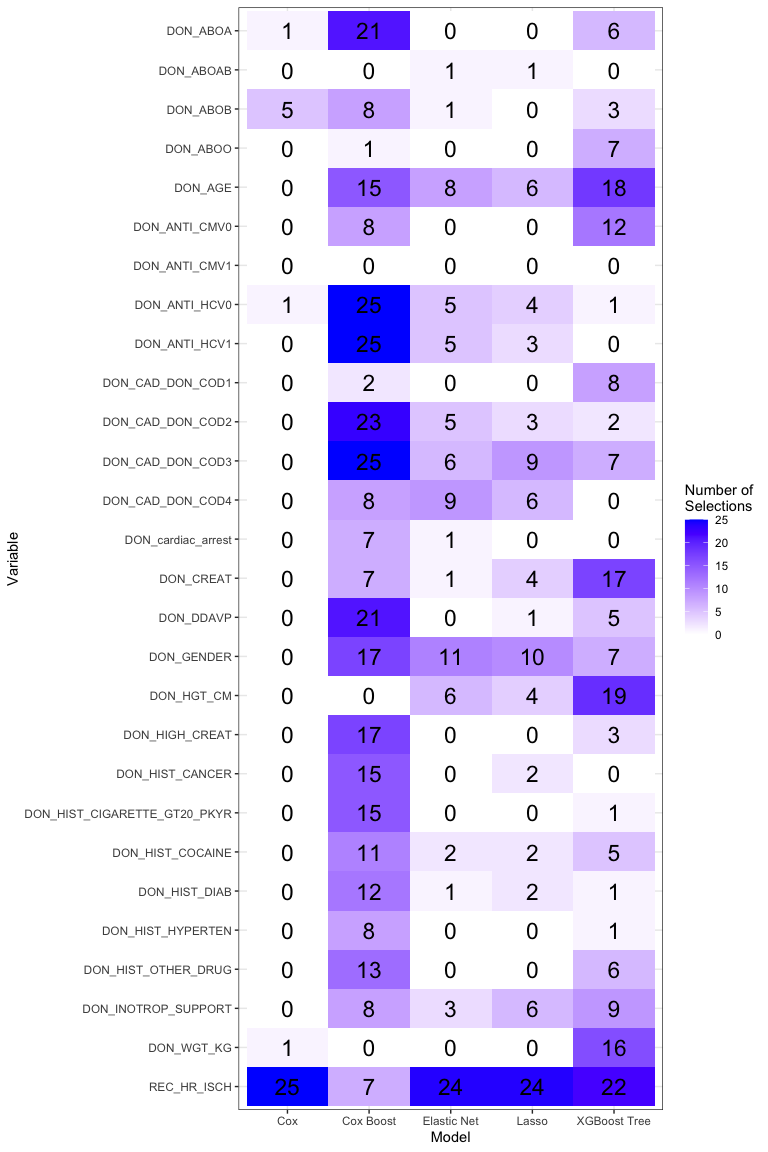** |
| **E**  **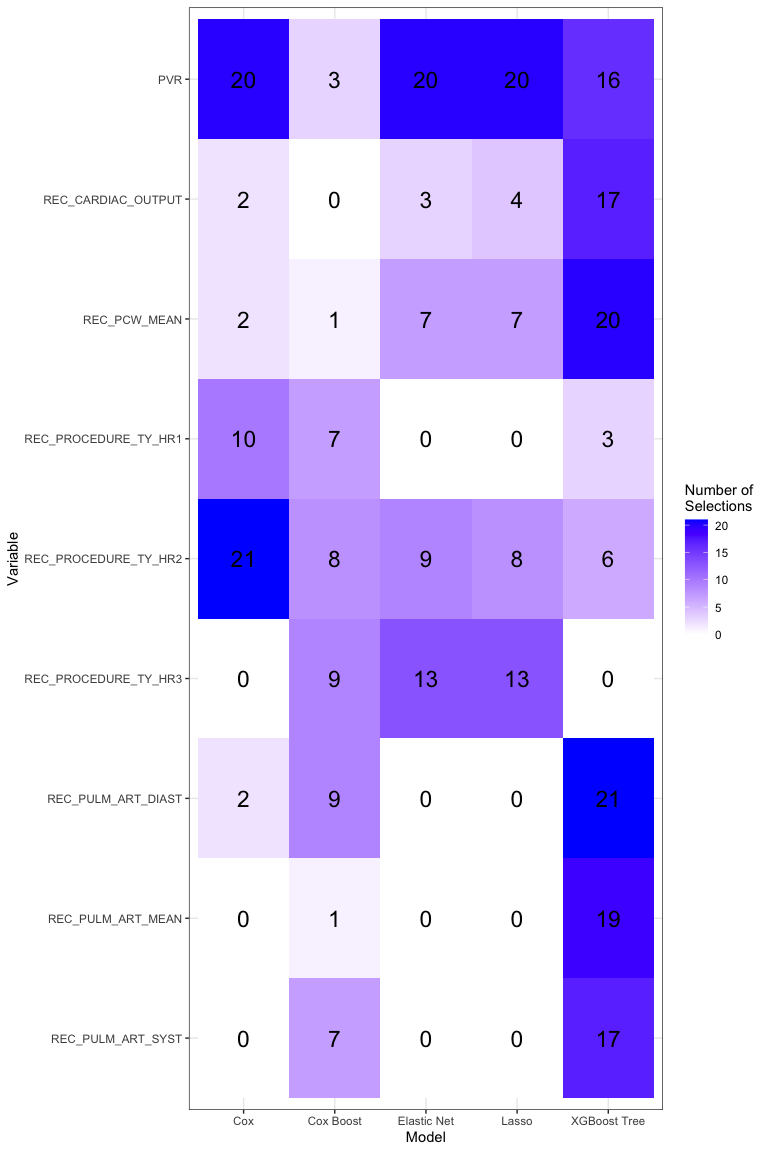** |

**Supplementary Figure 2.** Heatmaps of the total number of times a) demographic and clinical b) recipient c) candidate d) donor and e) procedural variables were selected by each model in the pre-policy cohort

| **A**  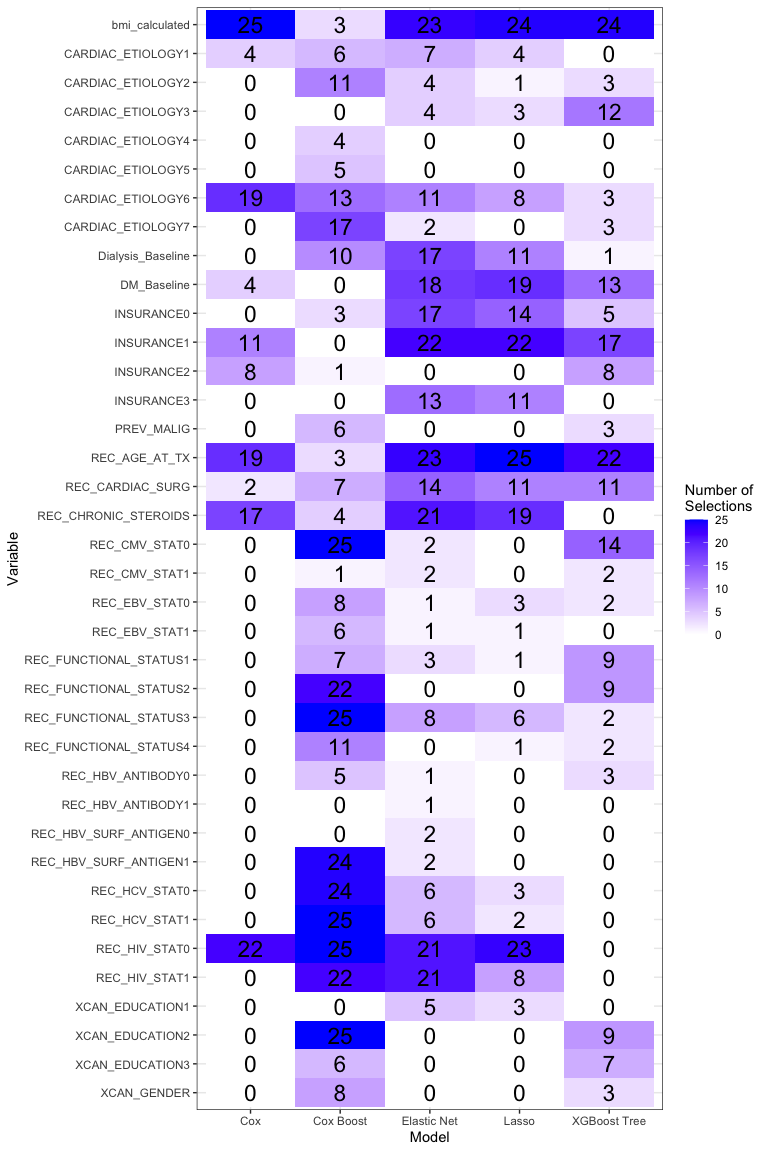 |
| --- |
| **B**  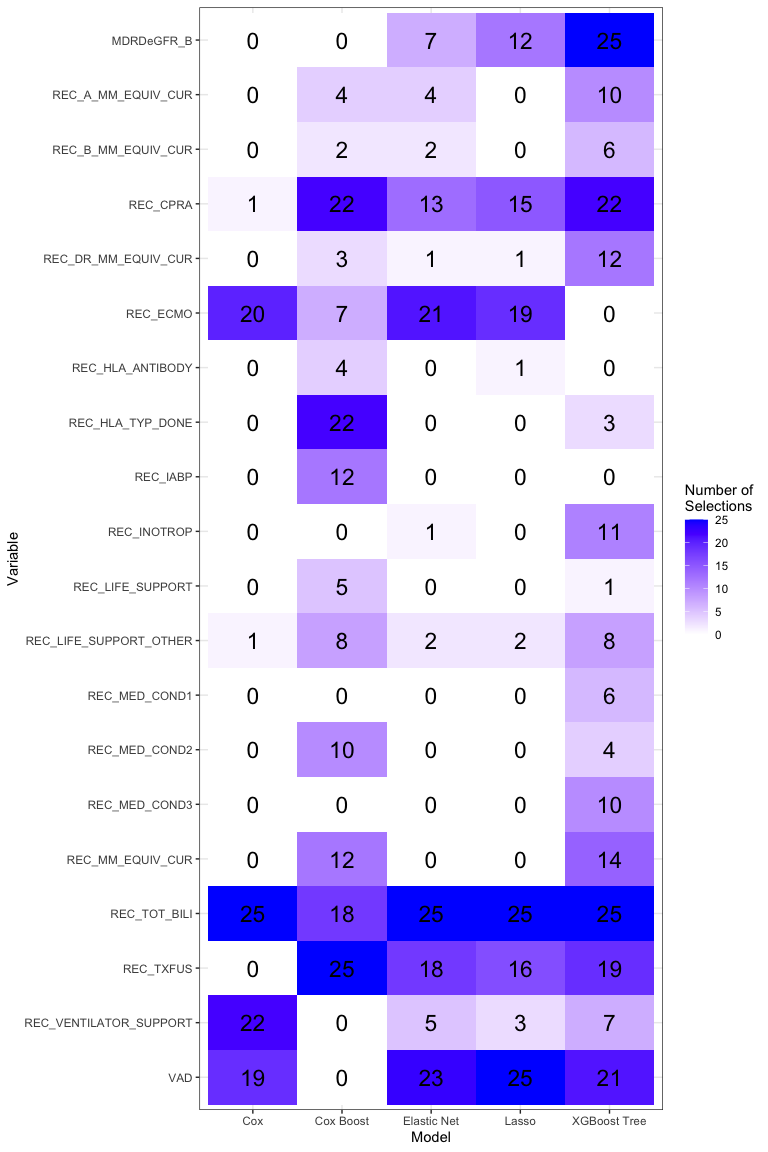 |
| **C**  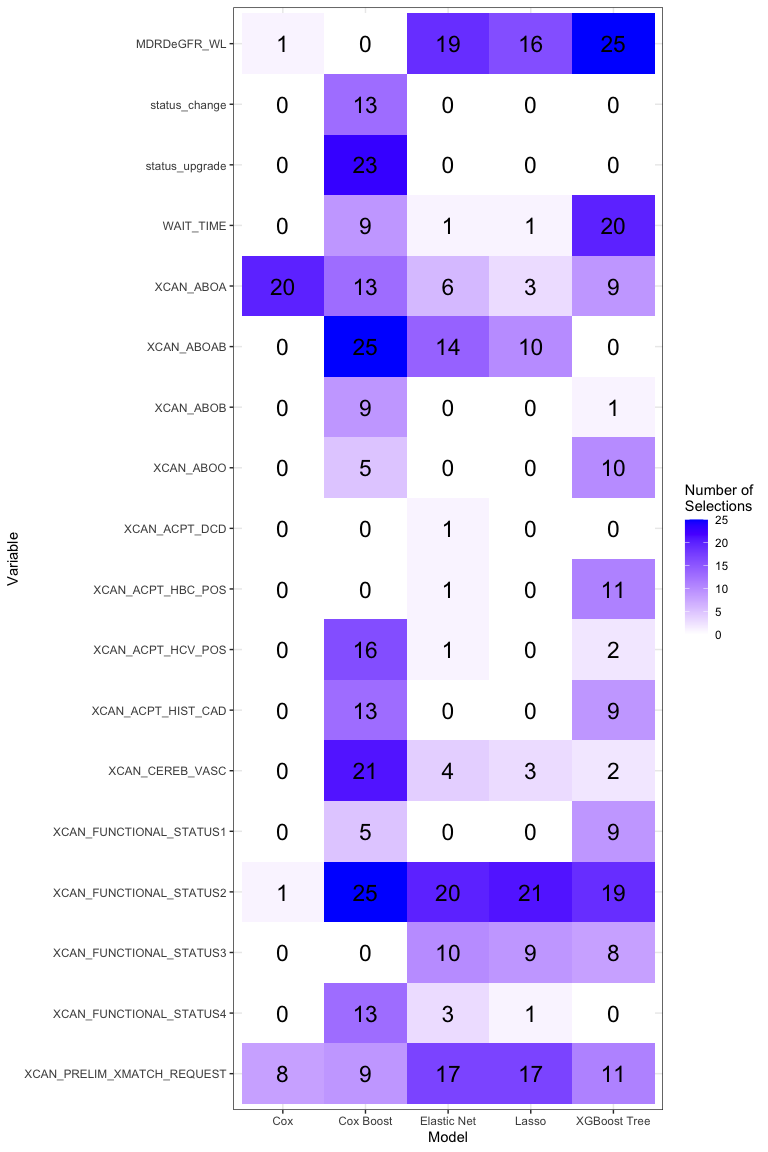 |
| **D**  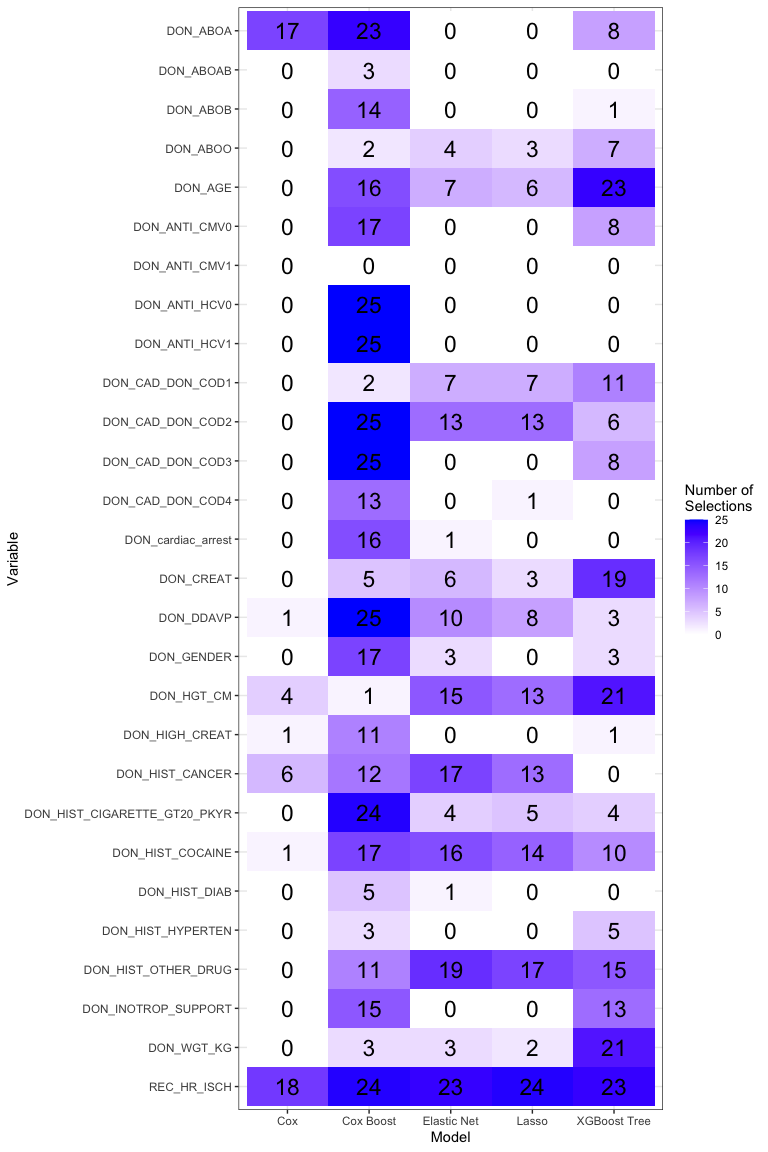 |
| **E**  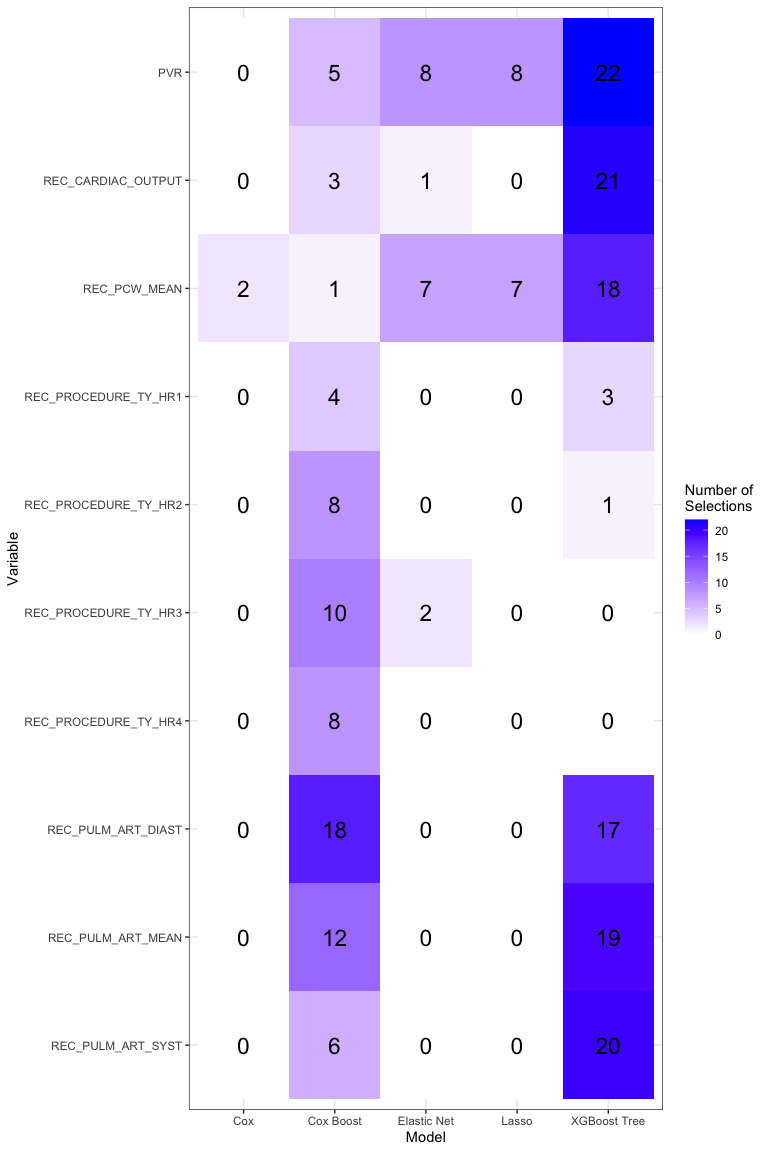 |

**Supplementary Figure 3.** Boxplots of hazard ratios of all a) demographic and clinical b) recipient c) candidate d) donor and e) procedural variables from Ridge in the post-policy cohort

| **A**  **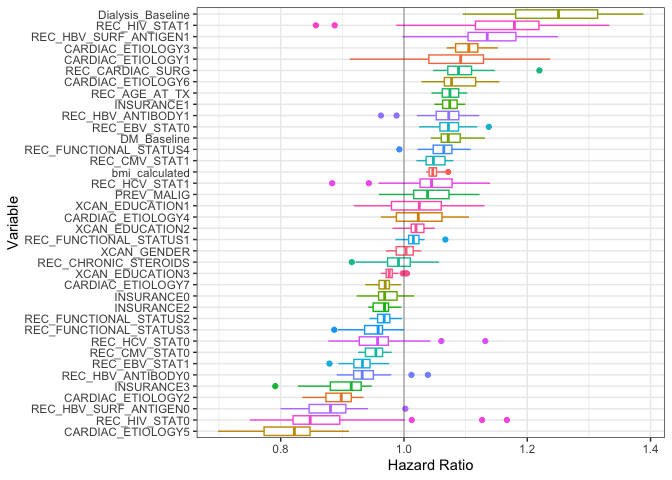** |
| --- |
| **B**  **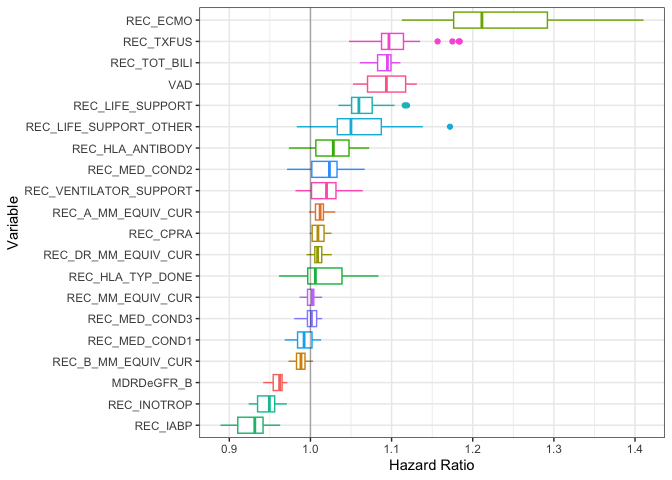** |
| **C**  **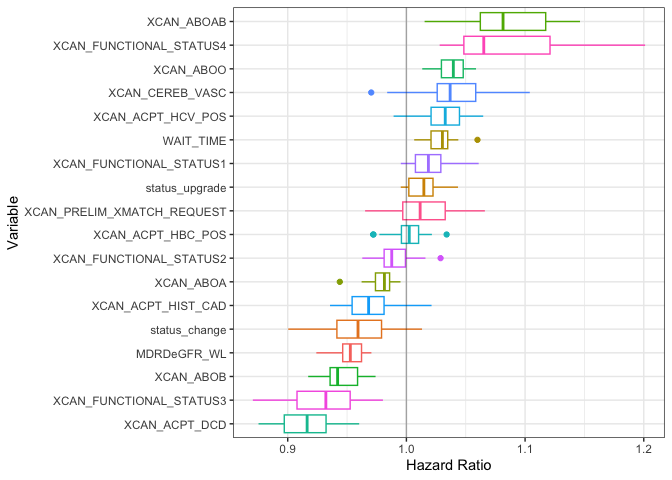** |
| **D**  **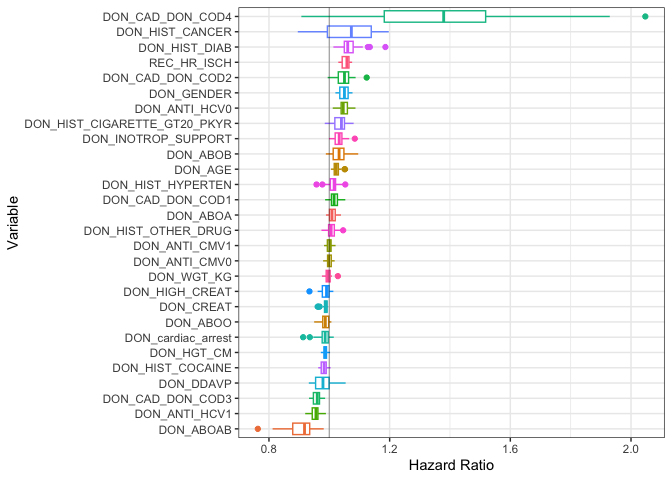** |
| **E**  **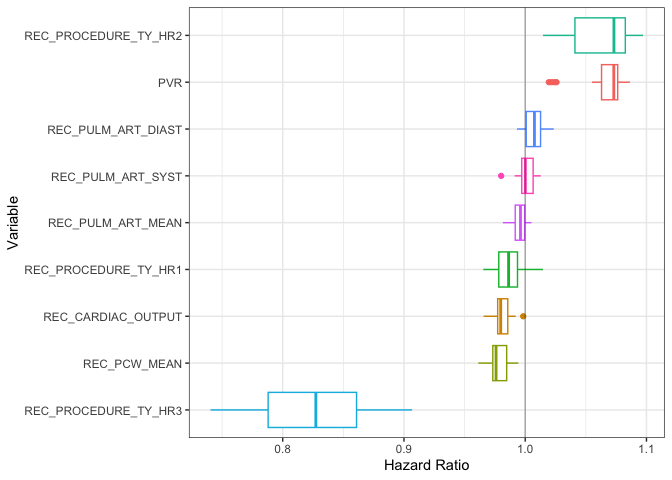** |

**Supplementary Figure 4.** Boxplots of hazard ratios of all a) demographic and clinical b) recipient c) candidate d) donor and e) procedural variables from Lasso in the post-policy cohort

| **A**  **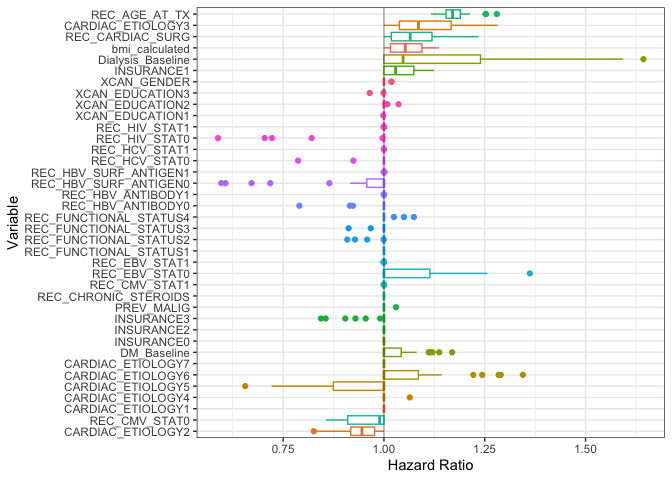** |
| --- |
| **B**  **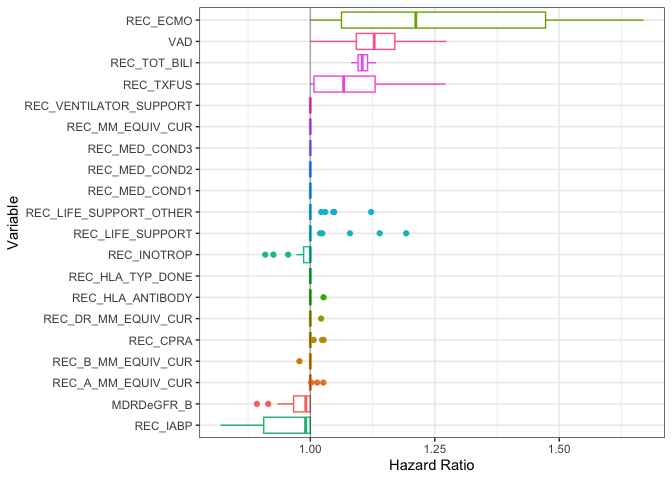** |
| **C**  **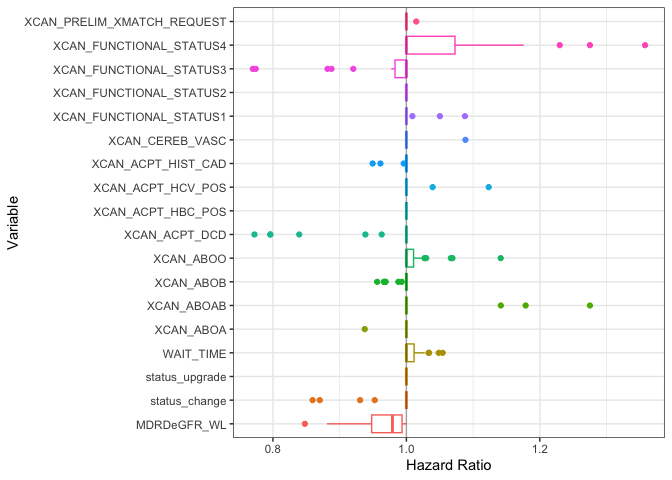** |
| **D**  **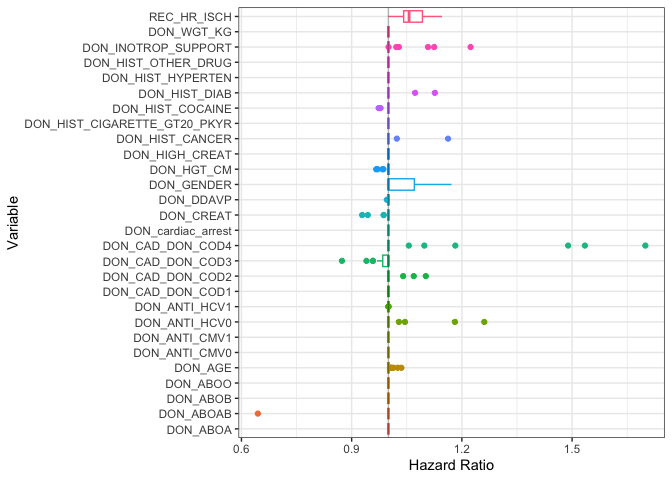** |
| **E**  **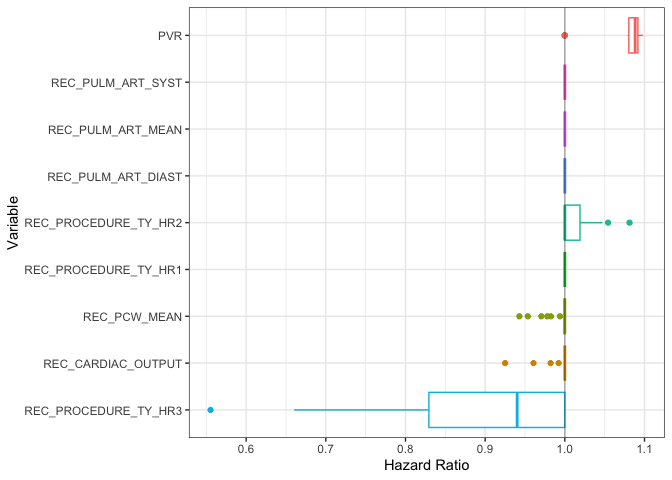** |

**Supplementary Figure 5.** Boxplots of hazard ratios of all a) demographic and clinical b) recipient c) candidate d) donor and e) procedural variables from Elastic Net in the post-policy cohort

| **A**  **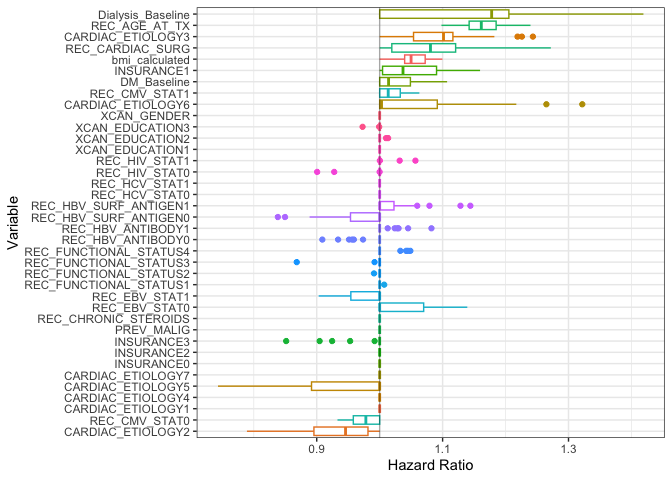** |
| --- |
| **B**  **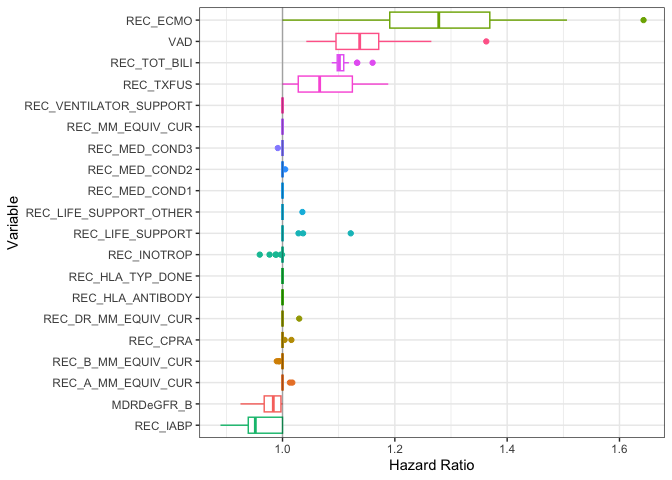** |
| **C**  **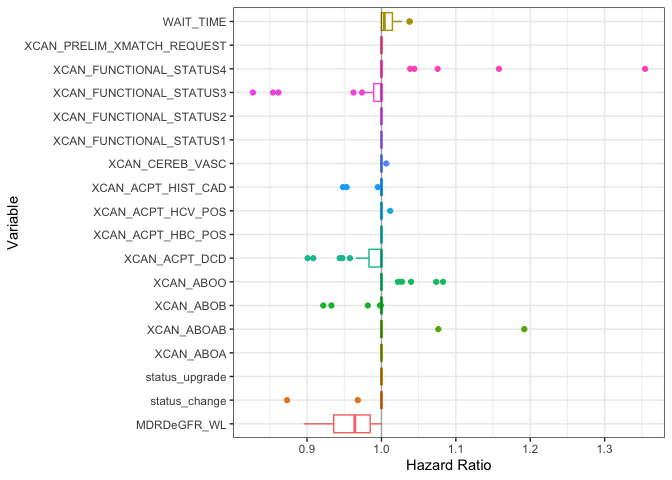** |
| **D**  **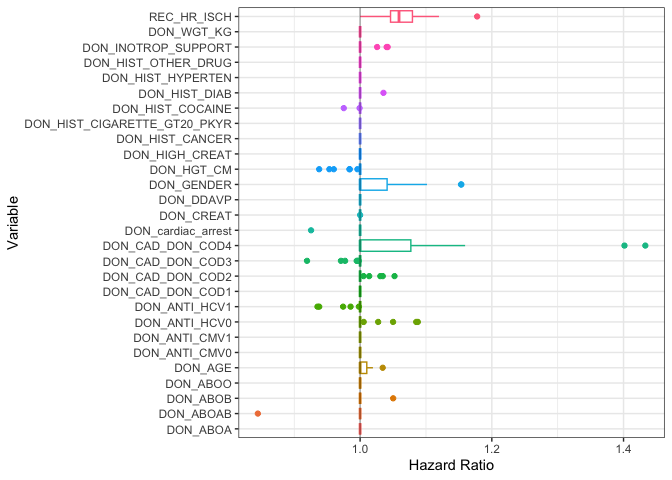** |
| **E**  **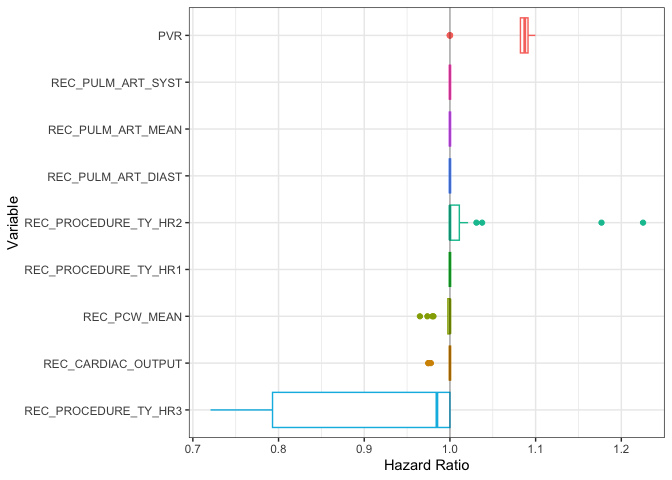** |

**Supplementary Figure 6.** Boxplots of importance of all a) demographic and clinical b) recipient c) candidate d) donor and e) procedural variables from Cox Boost in the post-policy cohort

| **A**  **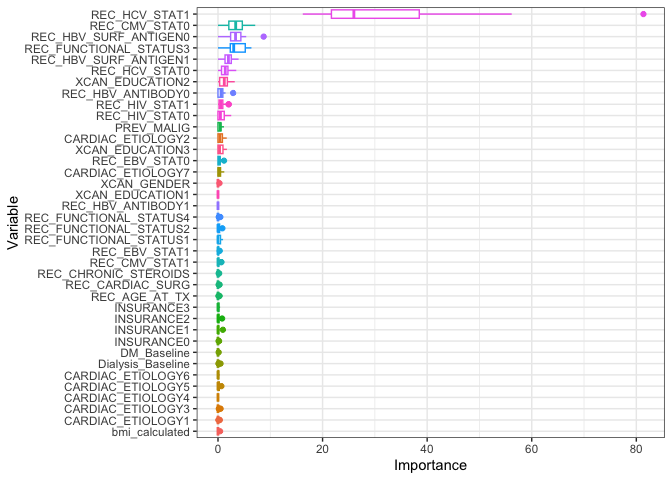** |
| --- |
| **B**  **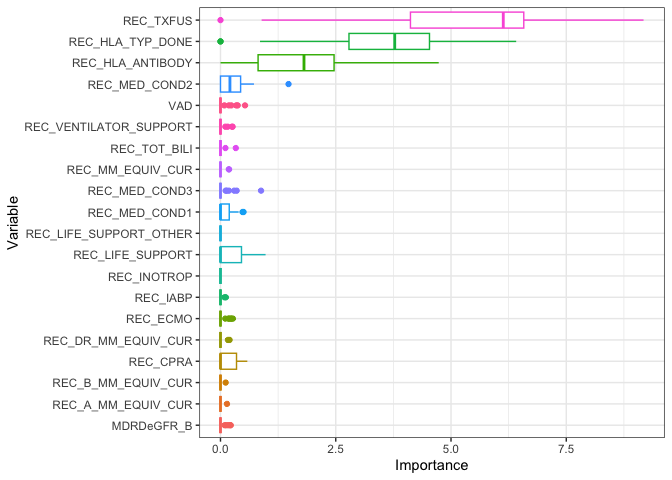** |
| **C**  **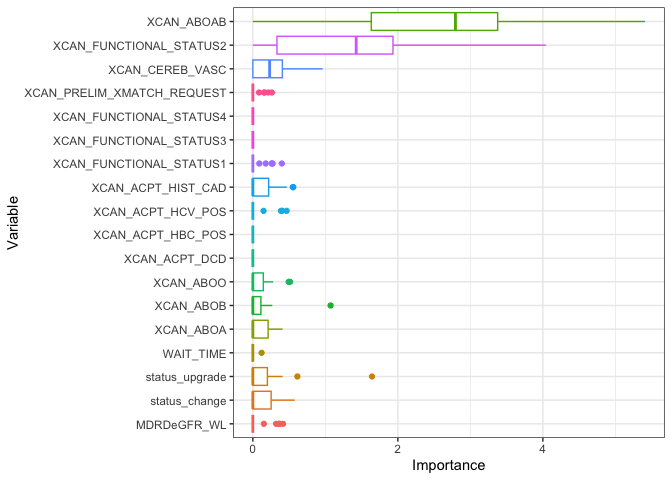** |
| **D**  **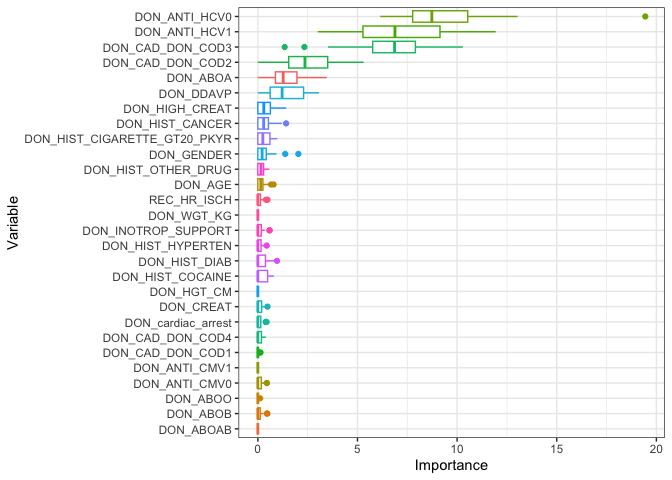** |
| **E**  **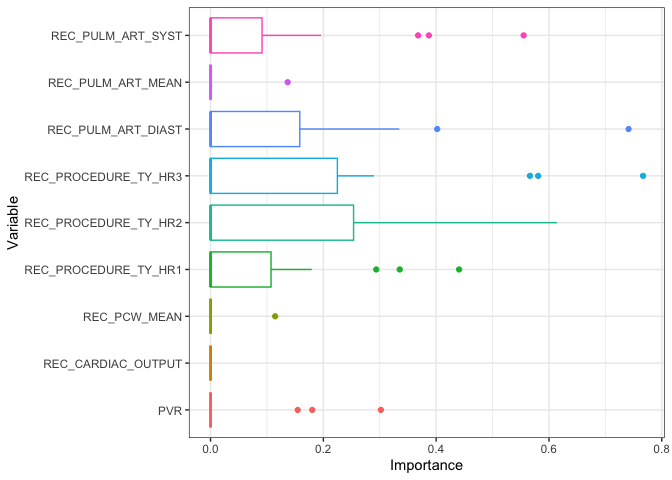** |

**Supplementary Figure 7.** Boxplots of importance of all a) demographic and clinical b) recipient c) candidate d) donor and e) procedural variables from XGBoost Linear in the post-policy cohort

| **A**  **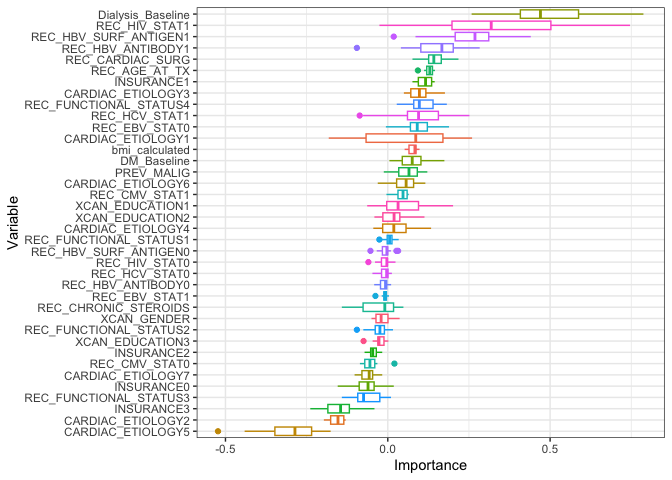** |
| --- |
| **B**  **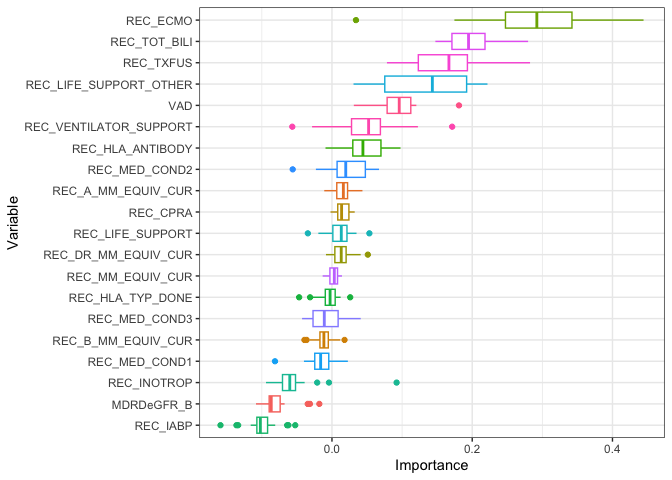** |
| **C**  **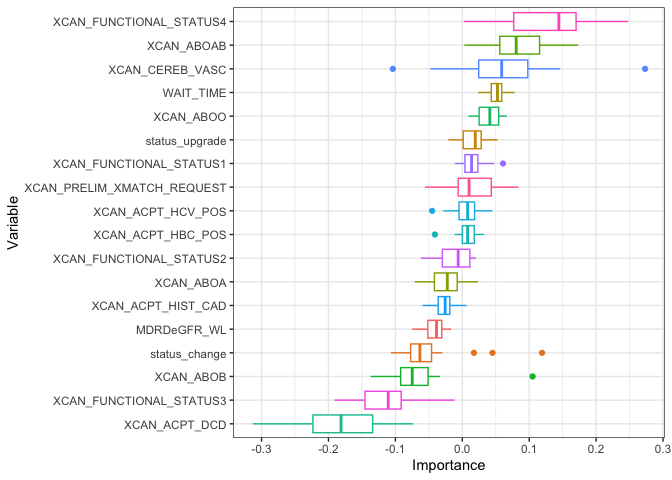** |
| **D**  **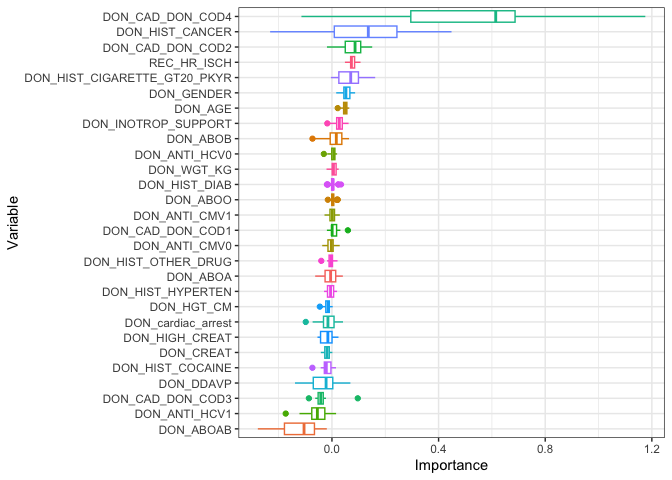** |
| **E**  **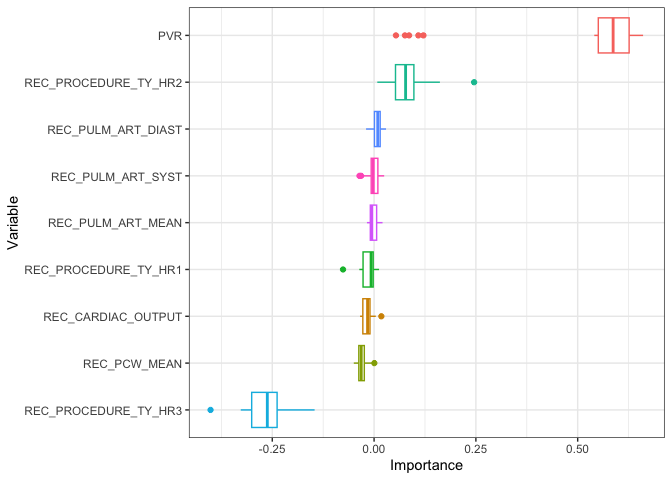** |

**Supplementary Figure 8.** Boxplots of weights of all a) demographic and clinical b) recipient c) candidate d) donor and e) procedural variables from XGBoost Tree in the post-policy cohort

| **A**  **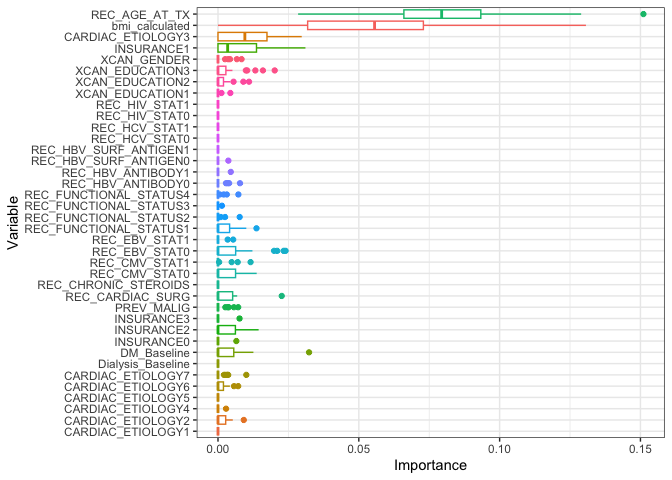** |
| --- |
| **B**  **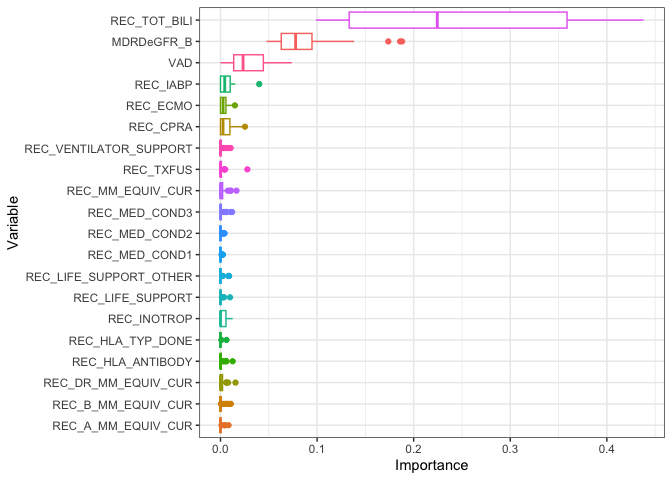** |
| **C**  **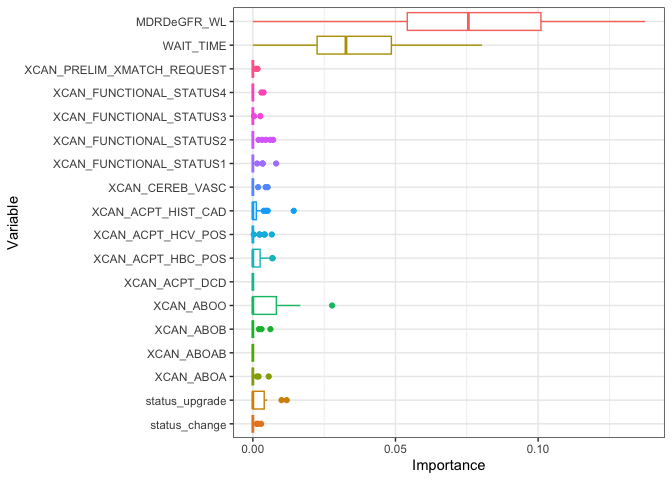** |
| **D**  **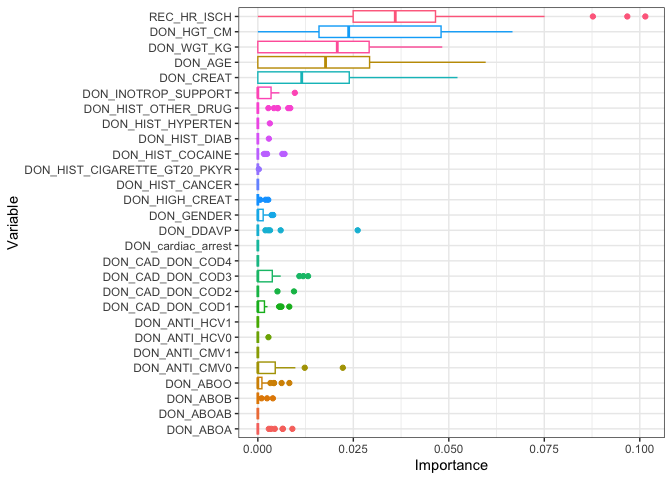** |
| **E**  **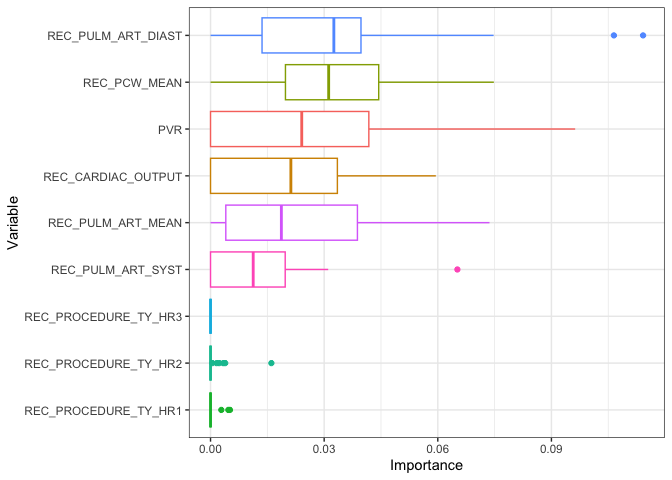** |

**Supplementary Figure 9.** Boxplots of weights of all a) demographic and clinical b) recipient c) candidate d) donor and e) procedural variables from Random Survival Forest in the post-policy cohort

| **A**  **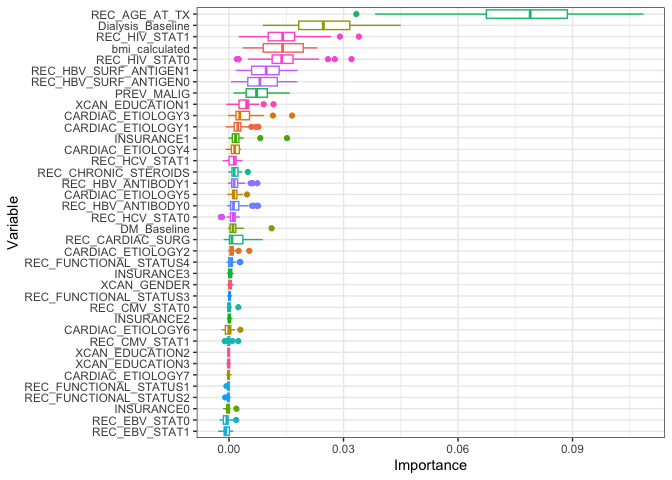** |
| --- |
| **B**  **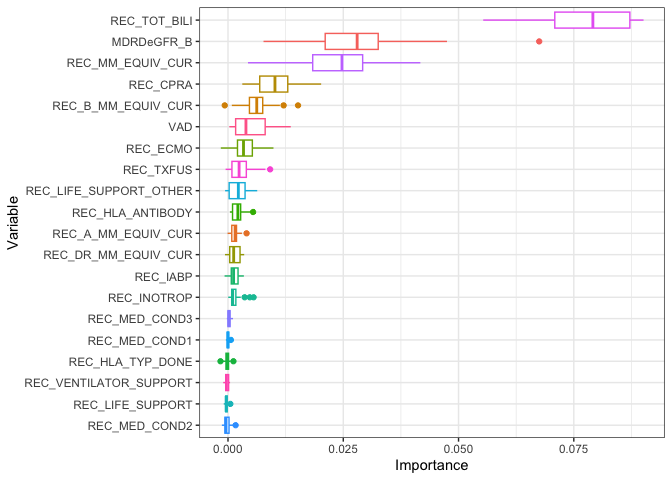** |
| **C**  **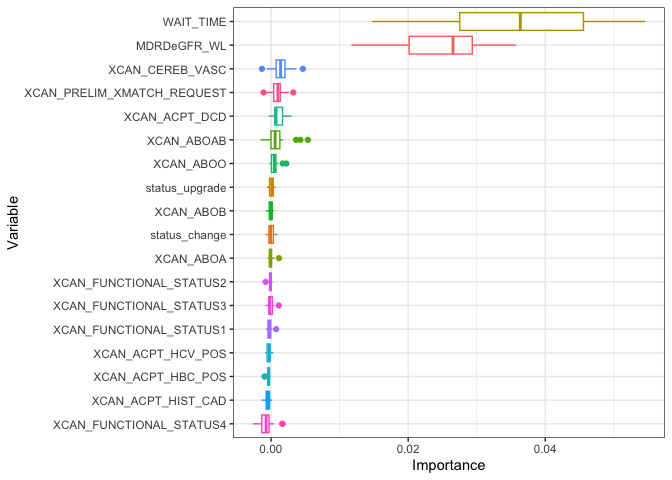** |
| **D**  **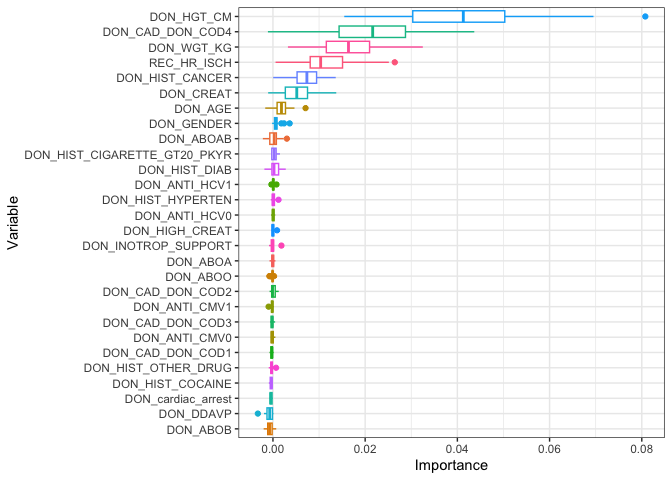** |
| **E**  **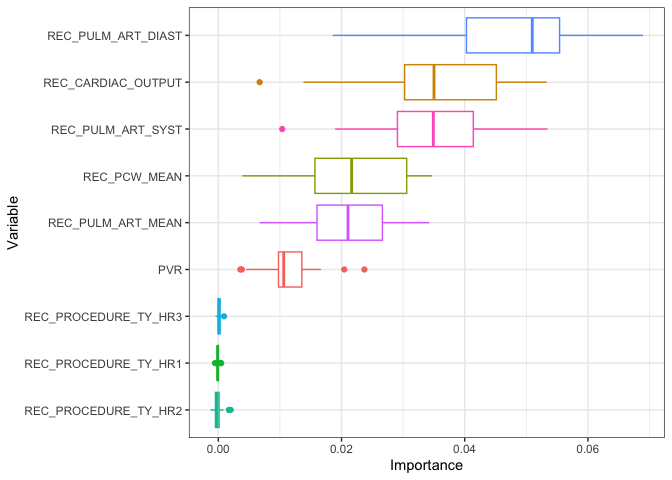** |
