# Supplementary material for: Survival machine learning methods for mortality prediction after heart transplantation in the contemporary era
Source: PLoS One. 2025 Jan 7;20(1):e0313600. doi: 10.1371/journal.pone.0313600 (PMC11706460; doi:10.1371/journal.pone.0313600)
Supplement: Attachment — Submitted filename: Lathan Liou SRTR ML Response to Reviewers Plos One.docx [file pone.0313600.s002.docx]

**PONE-D-24-16546**

**Survival Machine Learning Methods for Mortality Prediction After Heart Transplantation in the Contemporary Era**

Reviewer #1: Thanks for this important contribution. It might be worthwhile explaining the C-statistic in more detail - especially for non-experts. This exploration of ML in this space was first carried out by our group avout 10 years ago and did not see the light of publication. So, here's hoping you will.

- We thank Reviewer #1 for their encouraging comments. We have added in our Methods, page 8 more accessible language describing the C-statistic in more detail: “A C-index of 1 means the predictions were perfect: higher-risk patients are ranked ahead of lower-risk patients. A C-index of 0.5 means the predictions are random: half the time higher-risk patients are correctly ranked ahead, half the time lower-risk patients are incorrectly ranked ahead.”

Reviewer #2: The study's comprehensive analysis of survival machine learning algorithms and its potential clinical implications make it a significant addition to the literature. The authors have effectively addressed the key research questions and provided a well-structured presentation of their findings.
The manuscript is technically sound, with appropriate statistical analysis, and the data fully support the conclusions. The presentation is clear and intelligible, written in standard English. Therefore, it is recommended to be accepted for publication without the need for a review.

- We thank Reviewer #2 for their encouraging comments towards publication acceptance.
